# Frequency-dependent exacerbation of Alzheimer’s disease neuropathophysiology

**DOI:** 10.1038/s41598-019-44964-z

**Published:** 2019-06-20

**Authors:** Ksenia V. Kastanenka, Maria Calvo-Rodriguez, Steven S. Hou, Heng Zhou, Shuko Takeda, Michal Arbel-Ornath, Amanda Lariviere, Yee Fun Lee, Alex Kim, Jonathan M. Hawkes, Robert Logan, Danielle Feng, Xiqun Chen, Stephen N. Gomperts, Brian J. Bacskai

**Affiliations:** Department of Neurology, MassGeneral Institute of Neurodegenerative Diseases, Massachusetts General Hospital and Harvard Medical School, 114 Sixteenth St., Charlestown, MA 02129 USA

**Keywords:** Neural circuits, Alzheimer's disease

## Abstract

Neuronal activity patterns are disrupted in neurodegenerative disorders, including Alzheimer’s disease (AD). One example is disruption of corticothalamic slow oscillations responsible for sleep-dependent memory consolidation. Slow waves are periodic oscillations in neuronal activity occurring at frequencies of <1 Hz. The power, but not the frequency of slow oscillations is altered in a mouse model of AD. Optogenetic rescue of slow oscillations by increasing activity in cortical pyramidal neurons at the frequency of slow waves restores slow wave power, halts deposition of amyloid plaques and prevents neuronal calcium dysregulation. Here we determined whether driving this circuit at an increased rate would exacerbate the amyloid-dependent calcium dyshomeostasis in transgenic mice. Doubling the frequency of slow waves for one month with optogenetics resulted in increased amyloid beta - dependent disruptions in neuronal calcium homeostasis and loss of synaptic spines. Therefore, while restoration of physiological circuit dynamics is sufficient to abrogate the progression of Alzheimer’s disease pathology and should be considered an avenue for clinical treatment of AD patients with sleep disorders, pathophysiological stimulation of neuronal circuits leads to activity - dependent acceleration of amyloid production, aggregation and downstream neuronal dysfunction.

## Introduction

Disruptions in neuronal activity patterns have been reported in human patients and animal models of Alzheimer’s disease (AD)^1^. For instance, neuronal circuitry driving global cortical activity, such as slow oscillations, exhibits aberrant activity^2–4^. Slow waves are spontaneous thalamo-cortical oscillations occurring at frequencies less than 1 Hz. Slow oscillations alternate between up and down neuronal states. Up states are elicited by depolarizations of membrane potentials, while down states are characterized by neuronal hyperpolarizations. Cortical neuronal activity is responsible for state transitions^5^. Layer 5 pyramidal neurons generate waves through recurrent excitatory connections. Inhibitory interneurons synchronize that activity^6–8^. Voltage-sensitive dye imaging (VSDs) through cranial windows in anesthetized mice has allowed visualization of slow oscillations as waves propagating throughout the cortex^9,10^. Slow waves mediate a number of different processes including sleep-dependent memory consolidation^11,12^, which is disrupted in AD^13^.

Slow oscillations are altered in AD patients and animal models of AD early in the disease progression. Specifically, the power of slow waves is downregulated in patients with mild cognitive impairment (MCI) compared to healthy controls^3^. Low slow oscillation power has also been reported in a mouse model of amyloidosis (APP mice)^2^. Thus overproduction of Aβ disrupts slow oscillation activity, which leads to increased deposition of amyloid and neuronal calcium dysregulation. Restoring slow wave power at the normal frequency of 0.6 Hz by synchronizing cortical excitatory activity using light activation of Channelrhodopsin-2 (ChR2)-expressing pyramidal neurons stopped plaque deposition and prevented neuronal calcium elevations (calcium overload), characteristic of this animal model^2^. In addition, restoration of other oscillatory patterns attenuated amyloid load and memory impairment^14,15^. Thus restoration of oscillatory activity, including slow waves, might provide a potential venue for therapeutic development.

Since optogenetic restoration of pyramidal cell activity that rescued slow oscillations halted the progression of AD pathophysiology, it was imperative to determine whether the frequency of optogenetic stimulation was a defining factor. To answer this question, light activation of ChR2 targeted to cortical pyramidal neurons was used to increase the frequency of slow oscillations by a factor of two for a period of one month. Its effect on soluble Aβ levels, amyloid deposition, intraneuronal calcium levels and spine density was determined. Increasing the frequency of slow waves resulted in increased production of soluble Aβ, elevated amyloid plaque burden, increased number of neurons with calcium overload and decreased spine density on dendrites. Thus, while restoration of physiological circuit dynamics is sufficient to attenuate the progression of Alzheimer’s disease pathology and should be considered when devising treatments for AD, pathophysiological stimulation of neuronal circuits leads to activity-dependent acceleration of amyloid production, aggregation and downstream neuronal dysfunction.

## Results

### Doubling slow oscillation frequency elevates amyloidosis by increasing Aβ production

**Figure 1:**
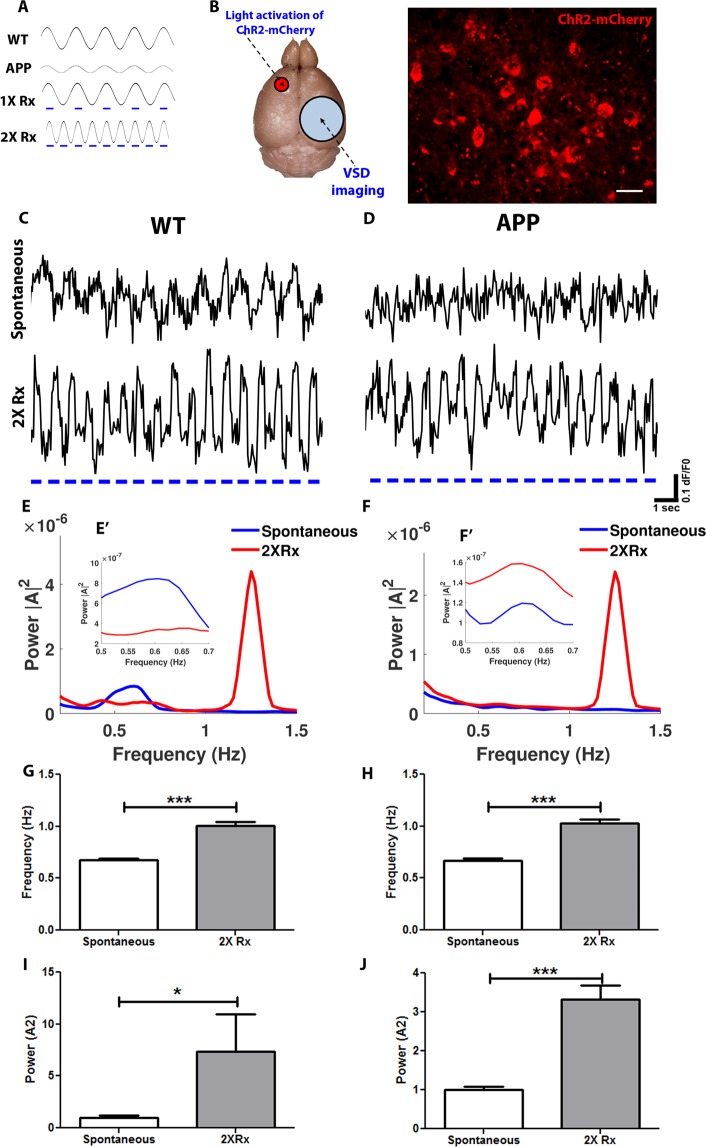
Use of optogenetics to manipulate slow oscillation frequency. (**A**) Schematic representations of slow oscillations in wildtype and APP mice (WT, APP) occurring spontaneously, during optogenetic stimulation at normal frequency (1X Rx), and during optogenetic stimulation at twice the normal frequency (2X Rx). Blue dashes represent light flashes at 473 nm. (**B**) *left*, schematic of mouse brain undergoing light stimulation of ChR2-mCherry expressed in anterior left hemisphere and voltage-sensitive dye (VSD) imaging through a cranial window over the somatosensory cortex in right hemisphere. *Right*, Expression of mCherry, a tag on AAV2-CamKII-ChR2-mCherry, in a mouse cortex. Scale bar, 30 µm. (**C**) Sample traces of spontaneous slow oscillations during VSD imaging from regions of interest (ROIs) in somatosensory cortex of an anesthetized 9 month old wildtype mouse before (Spontaneous) and during (2X Rx) optogenetic stimulation at twice the slow oscillation frequency. Blue dashes represent light flashes at 473 nm. (**D**) Sample traces of spontaneous slow oscillations during VSD imaging from regions of interest (ROIs) in somatosensory cortex of an anesthetized 9 month old APP mouse before (Spontaneous) and during optogenetic stimulation at twice the slow oscillation frequency (2X Rx). Note low power of spontaneous slow oscillations in the APP mouse compared to the wildtype. Blue dashes represent light flashes at 473 nm. (**E**) Power spectra density of slow oscillations in wildtype mice prior (Spontaneous) and during (2X Rx) light activation of ChR2 (n = 12–15 traces in 4 mice/group). ([A]^2^ = magnitude of Fourier amplitude squared). (**E’**) Power spectra insert from (**E**) for 0.5–0.7 Hz frequency range in wildtype mice. (**F**) Power spectra density of slow oscillations in APP mice prior (Spontaneous) and during (2X Rx) light activation of ChR2 (n = 13–14 traces in 3 mice/group). ([A]^2^ = magnitude of Fourier amplitude squared). (**F’**) Power spectra insert from (**F**) for 0.5–0.7 Hz frequency range in APP mice. (**G,H**) Mean slow oscillation frequency before (Spontaneous) and during (2X Rx) optogenetic manipulation in wildtype (**G**) and APP (**H**) mice (n = 3–4 mice/group). (**I,J**) Normalized slow oscillation power before (Spontaneous) and during (2X Rx) optogenetic manipulation (n = 3–4 mice/group). Data are shown as mean ± SEM. *p < 0.05, ***p < 0.001.

Optogenetics is a powerful tool to investigate neuronal activity disruptions in AD^16^. It allows targeting specific neuronal populations to increase or decrease neuronal firing probability. Slow oscillations are disrupted in an animal model of amyloidosis before substantial plaque deposition. Slow wave power is downregulated, while the frequency is maintained in APP mice compared to wildtype (WT) littermates (Fig. 1A, WT v.s. APP; Fig. 1E’,F’ spontaneous). Light activation of Channelrhodopsin-2 (ChR2)-expressing cortical pyramidal neurons at the normal frequency of slow oscillations, 0.6 Hz, restores slow oscillation power (Fig. 1A 1X Rx) and halts the progression of AD pathophysiology in APP mice^2^. However it was unclear whether restoration of normal circuit function was frequency-dependent. To that end the frequency of slow waves was experimentally manipulated.

Optogenetic approaches were used to increase slow oscillation frequency by a factor of two in APP mice and nontransgenic littermate controls (Fig. 1A 2X Rx). AAV2-CamKII-ChR2-mCherry (ChR2) was expressed virally in cortical excitatory neurons of APP and WT mice at 4 months of age (Fig. 1B left). ChR2 was injected into layer 5 left anterior cortex, while cranial windows were implanted over the right hemisphere (Fig. 1B left). Post-mortem cortical brain sections were used to verify expression of ChR2-mCherry (Fig. 1B right). Layer 5 cortical neurons are responsible for initiation of slow wave activity that spreads from one hemisphere to the other^5^. Voltage-sensitive dye (VSD) imaging with RH1691 was used to monitor slow wave activity with wide-field microscopy in mice anesthetized with 1–2% isoflurane. VSD imaging allowed monitoring slow oscillation circuit dynamics over time. Fourier transform analysis was performed to generate power spectral density plots (Fig. 1E,E’,F,F’) as well as determine the frequency and power of slow waves before (Spontaneous) and during the light treatment (2X Rx). Wildtype mice exhibited spontaneous slow oscillations at 0.6 Hz as reported previously (Fig. 1C top trace, Spontaneous; Fig. 1E,E’, Spontaneous)^2^. Light activation of ChR2 at 1.2 Hz increased cortical activity at that frequency, thus possibly doubling the slow oscillation frequency (Fig. 1C bottom trace, 2X Rx). APP mice exhibited disruptions in slow wave activity consistent with previous reports (Fig. 1D top trace, Spontaneous; Fig. 1F,F’, Spontaneous)^2,4^. Optogenetic stimulation of ChR2 at 1.2 Hz in APP mice doubled the frequency of slow oscillations (Fig. 1D bottom trace, 2X Rx). Fourier transform analysis revealed that the power and the frequency of slow waves was increased during optogenetic manipulations (Fig. 1E,F, n = 4 WT mice, n = 3 APP mice). The frequency of slow oscillations increased with optogenetic manipulation in WT and APP mice (Fig. 1G,H). The power of slow oscillations increased also (Fig. 1I,J; Supplementary Fig. 6). Furthermore, light stimulation of mCherry alone lacking ChR2 at 1.2 Hz failed to increase the slow wave frequency^2^.

The effect of optogenetic stimulation on slow oscillation frequency was also verified using local field potential (LFP) recordings with electrophysiology. Optogenetic stimulation significantly increased LFP power in the 1.2 Hz band (Supplementary Fig. 1).

**Figure 2:**
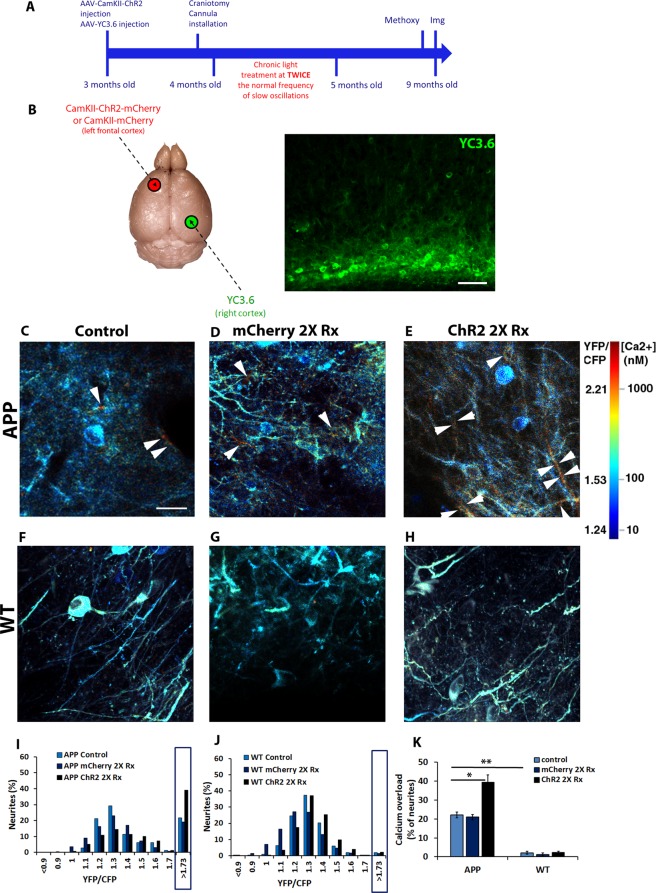
Doubling slow oscillation frequency increases neurite number with elevated levels of calcium in APP mice. (**A**) Timeline of the experiment. Methoxy-XO4 (Methoxy) injection was performed prior to the imaging session. (**B**) Schematic of mouse brain injected with CamKII-ChR2-mCherry virus in the left cortex and CBA-YC3.6 in the right cortex (left). YC3.6 expression in the mouse cortex (right). Scale bar, 40 µm. (**C–H**) *In vivo* multiphoton images of cortical neurites, pseudocolored according to [Ca^2+^]i, show the presence of elevated levels of calcium (yellow-red neurites) in 9 months old APP mice (**C**, arrowheads) in addition to neurites displaying normal calcium levels (for instance, blue neurites). Light activation of mCherry lacking ChR2 failed to increase the number of neurites with elevated levels of calcium (calcium overload) in 9 month old APP mice (**D**). Doubling slow oscillation frequency with light activation of ChR2 increased the number of neurites exhibiting calcium overload in 9 month old APP mice (**E**, arrowheads). 9 month old control wildtype mice (**F**) or wildtype mice that underwent light activation of mCherry lacking ChR2 exhibited neurites with normal calcium levels (**G**). Similarly 9 month old mice whose frequency of slow waves was sped up (**H**) exhibited neurites with normal levels of calcium. Scale bar, 20 µm. (**I**) Histograms comparing distributions of YFP/CFP ratios in neurites with YC3.6 in 9 month old control and treated APP mice (n = 4–5 mice/group). Calcium overload was defined as ratios greater than 2 standard deviations above the mean in wildtype mice (1.73). Boxed region shows percentages of neurites with calcium overload across conditions. (**J**) Distribution of neurite YFP/CFP ratios in control and treated wildtype mice (n = 4–11 mice/group). Boxed region shows percentages of neurites with calcium overload across conditions. (**K**) A bar graph showing the percentages of neurites exhibiting calcium overload across conditions at 9 months. Data are shown as mean ± SEM. *p < 0.05. **p < 0.01.

To determine the effect of doubling slow wave frequency on amyloid plaque burden (see Methods), APP mice expressing ChR2 unilaterally in anterior cortex were treated with light (4–8 mW, 473 nm blue light, 400 ms ON at 1.2 Hz) starting at 4 months. The treatment lasted for a month during which the mice were housed in microdialysis bowls where the animals could move freely with ad libitum access to food and water (Fig. 2A). At 9 months of age, the time point at which cortices exhibited substantial amyloid burden, cortices were imaged with multiphoton microscopy through cranial windows after intraperitoneal injection with methoxy-X04 to visualize amyloid plaques. As expected, control APP mice exhibited a robust amyloid plaque load within the cortex when imaged (Supplementary Fig. 2). However, mice whose frequency of slow waves was accelerated, exhibited an increase in amyloid plaque load compared to APP mice of same age (Supplementary Fig. 2). Light activation of mCherry alone failed to affect amyloid plaque load (Supplementary Fig. 2). The number of amyloid plaques (85 ± 6 plaques/mm^3^ Control, 85 ± 5 plaques/mm^3^ mCherry 2X Rx, 204 ± 17 plaques/mm^3^ ChR2 2X Rx, mean ± SEM; one way ANOVA followed by Dunn’s multiple comparison test, p < 0.001; n = 34 stacks in 4 control mice, n = 49 stacks in 5 mCherry expressing mice, n = 42 stacks in 4 ChR2 expressing mice) as well as amyloid plaque burden (8 ± 0.6% burden/mm^3^ Control, 8 ± 0.5% burden/mm^3^ mCherry expressing mice, 24 ± 2.4% burden/mm^3^ ChR2 expressing mice, mean ± SEM; one way ANOVA followed by Dunn’s multiple comparison test, p < 0.001; n = 34 stacks in 4 control mice, n = 49 stacks in 5 mCherry expressing mice, n = 42 stacks in 4 ChR2 expressing mice) was statistically higher in APP mice optogenetically treated with light (Supplementary Fig. 2). Thus, doubling the frequency of slow oscillations, increased amyloid plaque load in the cortices of APP mice.

Increases in synaptic activity using exogenous stimulation approaches have been implicated in regulating production of Aβ^17,18^. Additionally, endogenous Aβ fluctuations follow the circadian rhythm due to fluctuations in endogenous neuronal activity levels^19^. To determine whether production of Aβ is altered with twofold increases in the frequency of global cortical activity, we increased the slow oscillation frequency using optogenetics in one hemisphere and sampled cortical interstitial fluid (ISF) with microdialysis in the contralateral hemisphere. AAV2-CamKII-ChR2-mCherry was expressed in the anterior right cortex and a microdialysis probe was implanted in the left hemisphere (Supplementary Fig. 3). ISF Aβ 40 levels were measured with ELISA and compared prior, during and subsequent to brief stimulation in wildtype and APP mice (Supplementary Fig. 3). Optogenetic increases in the frequency of slow oscillations resulted in elevations of basal ISF Aβ 40 levels in wildtype as well as APP animals (Supplementary Fig. 3, Mann Whitney comparison to baseline at −2 h, 3–6 samples/group). Aβ levels returned to baseline levels after light stimulation ceased (Supplementary Fig. 3, Mann Whitney comparison to baseline at −2 h, 3–6 samples/group). Thus, doubling the frequency of slow waves leads to elevations of soluble Aβ 40 levels, resulting from increased Aβ production and might account for the increased amyloid plaque load (Supplementary Fig. 2).

### Elevating slow oscillation frequency exacerbates neuronal calcium overload

In addition to exhibiting elevations in soluble Aβ levels and amyloid plaque burden, adult APP mice have elevated levels of calcium, or calcium overload, in a fraction of vulnerable cortical neurons^20^. This calcium overload is indicative of disrupted calcium homeostasis and aberrant neuronal activity. To determine whether doubling slow wave frequency leads to additional neurons with calcium overload, we expressed the radiometric calcium sensor, yellow cameleon 3.6 (YC3.6), in APP mice and nontransgenics^21^ (Fig. 2A,B). We defined calcium overload as a YFP/CFP ratio amounting to 2 standard deviations above the average ratio of all wildtype mouse neurons, >1.73 for neuronal processes (neurites). At 9 months of age, APP mice exhibited calcium overload in a fraction of their neurites (Fig. 2C, arrowheads; I, boxed values; 22 ± 1%, n = 1123 neurites in 5 APP control mice). Increasing the frequency of slow waves with light led to an increase in the percentage of neurites with calcium overload (Fig. 2E, arrowheads; I, K, 39 ± 2%, n = 1121 neurites in 4 APP ChR2 expressing mice; p < 0.05, Kruskal-Wallis test followed by Dunn’s Multiple comparison test). To determine whether optogenetic treatment leads to calcium dyshomeostasis in nontransgenic mice, these animals also underwent light treatment. 9 month old wildtype mice exhibited a low percentage of neurites with calcium overload (Fig. 2F,J, 2 ± 1% WT control vs 22 ± 1% APP control, p < 0.01, Kruskal-Wallis test followed by Dunn’s Multiple comparison test, n = 2620 neurites in 11 WT control mice). That percentage remained low after light activation of ChR2 (Fig. 2H,J,K, 1 ± 1%, n = 1412 neurites in 4 WT ChR2 expressing mice). To control for light toxicity, light treatment was performed in APP and WT animals expressing mCherry and lacking ChR2. The treatment failed to significantly increase the percentage of neurites with calcium overload above control levels (Fig. 2D,G,I–K, 22 ± 1% APP mCherry expressing vs 21 ± 4% APP control mice, n = 1230 neurites in 5 APP mCherry expressing mice; 2 ± 1% WT mCherry expressing vs 2 ± 1% WT control, n = 1412 neurites in 4 WT mCherry expressing mice; Kruskal-Wallis test followed by Dunn’s Multiple comparison test). Thus, doubling slow oscillations results in exacerbations of Aβ-dependent calcium dyshomeostasis in APP mice.

**Figure 3:**
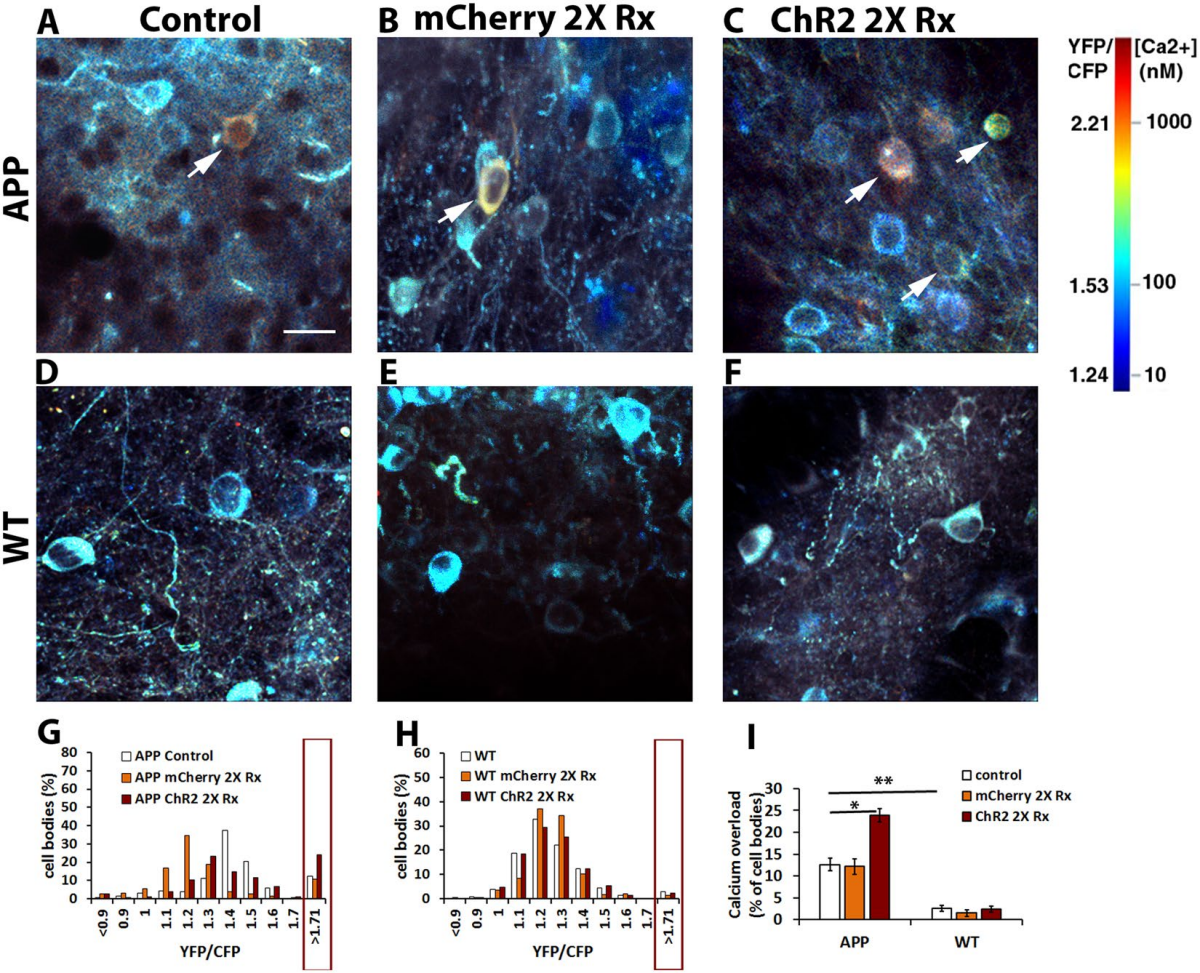
Increasing slow oscillation frequency elevates intracellular calcium in neuronal cell bodies of APP mice. (**A**–**F**) *in vivo* multiphoton images of cortical cell bodies, pseudocolored according to [Ca^2+^]i, show the presence of elevated levels of calcium (yellow-red cell bodies) in 9 months old APP mice (**A**, arrow) in addition to cell bodies displaying normal calcium levels (shown in blue). Light activation of mCherry lacking ChR2 at twice the frequency of slow waves failed to increase the number of neuronal somas with calcium overload (**B**, arrow). Doubling slow oscillation frequency with light activation of ChR2 increased the number of cell bodies exhibiting calcium overload in 9 month old APP mice (**C**, arrows). Control (**D**) or treated wildtype mice (**E,F**) exhibit neuronal somas with normal levels of calcium. Scale bar, 20 µm. (**G**) Histograms comparing distribution of YFP/CFP ratios in neuronal cell bodies with YC3.6 in control and treated APP mice (n = 4–5 mice/group). Calcium overload was defined as ratios greater than 2 standard deviations above the mean in wildtype mice (1.71). Boxed region shows percentages of cell bodies with calcium overload across conditions. (**H**) Distribution of cell body YFP/CFP ratios in control and treated wildtype mice (n = 4–11 mice/group). Boxed region shows percentages of cell bodies with calcium overload across conditions. (**I**) A bar graph showing the percentages of neuronal somas exhibiting calcium overload across conditions at 9 months. Data are shown as mean ± SEM. *p < 0.05. **p < 0.01.

Calcium is a ubiquitous ion, which is essential for a number of cellular functions, and it is regulated with some independence in different cellular compartments, particularly in neurons. In addition to assessment of calcium levels within neuronal processes, calcium levels were measured within neuronal somas. 9 month old APP mice exhibited calcium overload, or ratios of YFP/CFP >1.71, within a fraction of their cortical cell bodies (Fig. 3A,G, 13 ± 2%, n = 228 cell bodies in 5 APP control mice). Doubling slow oscillations frequency increased the percentage of neuronal cell bodies with calcium overload (Fig. 3C,G,I, 24 ± 1%, n = 182 cell bodies in 4 APP ChR2 expressing mice; p < 0.05 Kruskal-Wallis test followed by Dunn’s Multiple comparison test). Conversely to APP mice, wildtype controls of same age exhibited a small percentage of neurons with calcium overload (Fig. 3D,H,I, 3 ± 1% WT control v.s. 13 ± 2% APP control, p < 0.01 Kruskal-Wallis test followed by Dunn’s Multiple comparison test, n = 788 cell bodies in 11 WT control mice). Driving neuronal activity with light did not increase the percentage of cell bodies with elevated calcium levels in these animals (Fig. 3F,H,I; 2 ± 1%, n = 425 cell bodies in 4 WT ChR2 expressing mice, Kruskal-Wallis test followed by Dunn’s Multiple comparison test). Furthermore, there were no signs of light toxicity, since light treatment of animals expressing mCherry alone failed to elevate calcium in APP and WT mice above control levels (Fig. 3B,E,G–I, 12 ± 2% APP mCherry expressing v.s. 13 ± 2% APP control, n = 367 cell bodies in 5 APP mCherry expressing mice; 1 ± 1% WT mCherry expressing v.s. 3 ± 1% WT control, n = 424 cell bodies in 4 WT mCherry expressing mice; Kruskal-Wallis test followed by Dunn’s Multiple comparison test).

Thus, twofold increases in slow oscillation frequency led to intracellular calcium elevations within neurites and cell bodies in APP but not wildtype mice. Light stimulation alone was not a concern as it failed to elevate intracellular calcium.

### Increasing the frequency of slow oscillations decreases spine density

**Figure 4:**
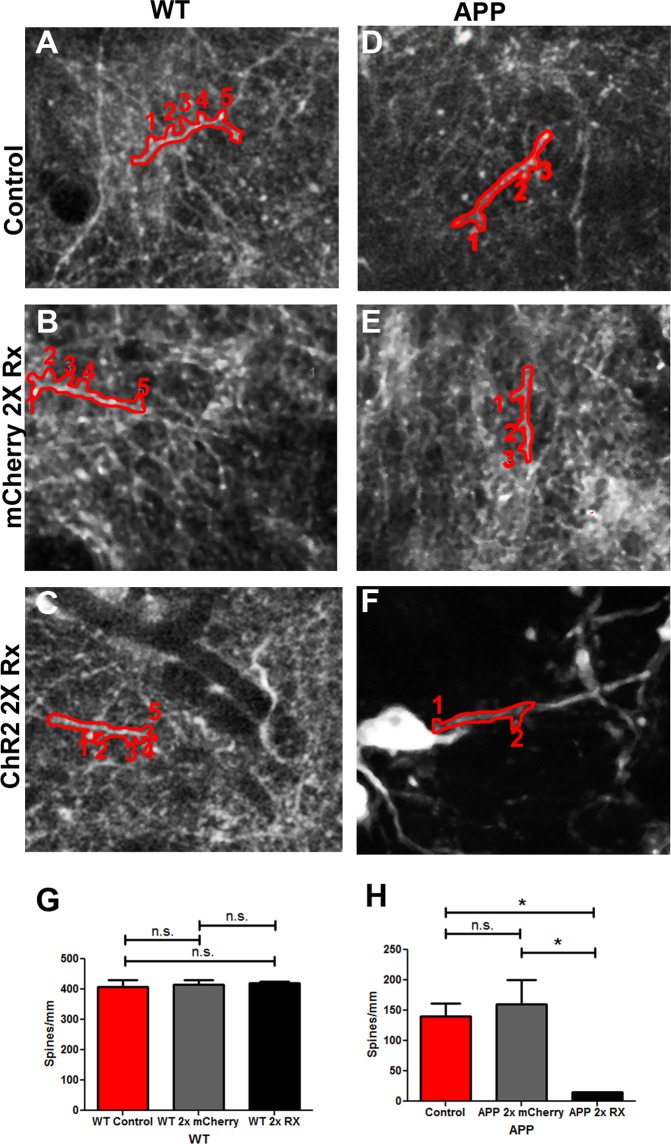
Doubling the frequency of slow waves decreases spine density. (**A**–**C**) Multiphoton images of cortical neurons, their processes and dendritic spines labeled with YC3.6 in control WT mice (**A**) and WT mice whose mCherry (**B**) or ChR2 (**C**) was treated with light at twice the frequency of slow waves (n = 4–7 mice/group). Individual neuronal processes and spines are outlined in red. Spines are numbered. (**D**–**F**) Multiphoton images of cortical neurons, their processes and dendritic spines labeled with YC3.6 in control APP mice (**D**) and APP mice expressing mCherry (**E**) or ChR2 (**F**) and treated with light at twice the frequency of slow waves (n = 4–6 mice/group). Individual neuronal processes and spines are outlined in red. Spines are numbered. Scale bar, 30 µm. (**G**) A bar graph comparing the spine density in wildtype mice and those treated with light. (**H**) A bar graph comparing the spine density in APP mice and those treated with light. Data are shown as mean ± SEM. *p < 0.05.

The number of dendritic spines and the number of synapses positively correlates with cognitive function^22,23^. Cortical dendrites of APP animals exhibit lower spine density, which is correlated with calcium overload, compared to wildtype controls^24,25^. To determine whether the number of dendritic spines is altered when the frequency of slow waves is increased, dendritic spines on cortical neurons were imaged with multiphoton microscopy in APP and WT mice (Fig. 4A–F). Spine density on neurite segments longer than 8 µm was included in the analysis. Consistent with previous reports, APP animals exhibited lower spine density compared to wildtype littermates (Fig. 4A,D). Specifically, APP cortical dendrites had 140 ± 22 spines/mm of dendrite compared to 409 ± 20 spines/mm of wildtype dendrite (Fig. 4A,D,G,H, n = 173 neurites in 7 control APP mice, n = 138 neurites in 4 control WT mice). Spine density was even lower in APP mice whose slow oscillation frequency was doubled (Fig. 4D,F,H, 140 ± 22 spines in APP control mice v.s. 15 ± 1 spines in APP ChR2 expressing mice, n = 107 neurites in 4 APP ChR2 expressing mice, p < 0.05, Kruskal-Wallis test followed by Dunn’s Multiple comparison test). Furthermore, light activation of mCherry alone failed to statistically alter spine density on APP dendrites (Fig. 4D,E,H, 140 ± 22 spines APP control v.s. 160 ± 41 spines APP mCherry expressing mice, n = 182 neurites in 6 APP mCherry expressing mice, Kruskal-Wallis test followed by Dunn’s Multiple comparison test). Thus, increasing the frequency of neuronal activity detrimentally affects neuronal spine density in APP mice. Interestingly, light treatment failed to significantly alter spine density in wildtype mice whether ChR2 was present or not (Fig. 4A–C,G, 409 ± 20 spines/mm of dendrite WT Control, 416 ± 15 spines/mm of dendrite WT mCherry expressing mice, 420 ± 5 spines/mm of dendrite WT ChR2 expressing mice, n = 138 dendrites in 4 WT Control mice, n = 143 neurites in 4 WT mCherry expressing mice, n = 174 neurites in 6 WT ChR2 expressing mice, Kruskal-Wallis test followed by Dunn’s Multiple comparison tests). Thus light stimulation of ChR2 or light alone did not alter cortical spine density in wildtype mice.

### Increasing the frequency of slow oscillations does not alter levels of excitatory or inhibitory neurotransmitters

Deficits in inhibitory elements of the slow oscillation circuit might be responsible for disruptions of slow wave activity, since GABA levels were downregulated in APP mice at 7 months. Furthermore, topical application of GABA restored the power of slow waves^2^. Here, the levels of GABA were assessed with immunohistochemistry and verified with HPLC in 9 month old animals. Similar to earlier stages of the disease progression, 9 month old APP mice continued exhibiting low cortical GABA levels compared to those of wildtype littermates (Supplementary Fig. 4, n = 156–340 ROIs in 4–9 mice/group, Kruskal-Wallis test followed by Dunn’s Multiple comparison test, p < 0.001). Increasing the frequency of slow waves failed to statistically alter GABA levels in APP and WT mice (Supplementary Fig. 4, n = 156–340 ROIs in 4–9 mice/group, Kruskal-Wallis test followed by Dunn’s Multiple comparison test). Light itself failed to alter GABA levels (Supplementary Fig. 4, n = 156–340 ROIs in 4–9 mice/group, Kruskal-Wallis test followed by Dunn’s Multiple comparison test). Low cortical GABA levels in APP mice compared to nontransgenics were verified with HPLC measurements (Supplementary Fig. 4, n = 4–11 mice/group, Kruskal-Wallis test followed by Dunn’s Multiple comparison test, p < 0.05).

Levels of the excitatory neurotransmitter glutamate, important for generation of slow oscillations, were also assessed. Cortical glutamate levels were similar in APP and WT animals at 7 months of age^2^. Interestingly, 9 month old APP mice exhibited reduced levels of glutamate compared to those in wildtype littermate controls (Supplementary Fig. 4, n = 160–318 ROIs in 4–9 mice/group, Kruskal-Wallis test followed by Dunn’s Multiple comparison test, p < 0.001). This suggested a temporal progression of cortical circuitry disruption, inhibitory followed by excitatory. Doubling slow wave frequency failed to statistically alter glutamate levels in APP and WT mice (Supplementary Fig. 4, n = 160–318 ROIs in 4–9 mice/group, Kruskal-Wallis test followed by Dunn’s Multiple comparison test). Furthermore, light activation of mCherry did not alter glutamate levels (Supplementary Fig. 4, n = 160–318 ROIs in 4–9 mice/group, Kruskal-Wallis test followed by Dunn’s Multiple comparison test). Low cortical glutamate levels in APP mice compared to wildtype littermates were verified with HPLC measurements (Supplementary Fig. 4, n = 4–15 mice/group, Kruskal-Wallis test followed by Dunn’s Multiple comparison test, p < 0.05).

Therefore, this data underscores the fact that AD is a truly progressive disease, since disruptions in cortical circuitry starts with deficits in GABA followed by those in glutamate.

### Cortical slow wave activity does not alter expression of receptors mediating inhibitory neurotransmission

7 month old APP mice exhibit downregulations in GABA_A_ and GABA_B_, the ionotropic and metabotropic receptors mediating inhibitory neurotransmission in the cortex^2^. Thus the protein levels of these receptors were assessed with immunohistochemistry. Interestingly, GABA_A_ receptor expression was reduced in 9 month old APP animals compared to WTs (Supplementary Fig. 5, n = 160–279 ROIs in 4–9 mice/group, Kruskal-Wallis test followed by Dunn’s Multiple comparison test, p < 0.05). Similarly GABA_B_ receptor expression was reduced in transgenics compared to controls (Supplementary Fig. 5, n = 160–279 ROIs in 4–9 mice/group, Kruskal-Wallis test followed by Dunn’s Multiple comparison test). Increasing the frequency of slow waves failed to statistically alter the levels of these receptors in APP and WT mice (Supplementary Fig. 5, n = 160–279 ROIs in 4–9 mice/group, Kruskal-Wallis test followed by Dunn’s Multiple comparison test). Furthermore, light itself did not significantly alter receptor levels in these animals (Supplementary Fig. 5, n = 160–279 ROIs in 4–9 mice/group, Kruskal-Wallis test followed by Dunn’s Multiple comparison test). Thus in addition to excitatory and inhibitory neurotransmitter deficits, APP animals continued exhibiting deficits in receptors mediating inhibitory neurotransmission. Optogenetic increases in the frequency of slow oscillation activity failed to alter the receptor levels.

## Discussion

Neuronal activity patterns are disrupted in AD^26^. One example is aberrant slow wave activity^2–4^. Restoring slow wave power to normal levels, while maintaining the frequency at 0.6 Hz, slowed the progressive pathophysiology associated with AD^2^. Specifically, it halted amyloidosis and calcium dysregulation in an AD animal model. To determine how important the specific frequency of neuronal activity is when aiming to slow the disease progression, we manipulated slow oscillation frequency. Optogenetics is a powerful tool to drive neuronal activity. Thus, we used optogenetics to increase the slow wave frequency to 1.2 Hz in APP mice to determine its effect on the progression of the pathophysiology. Slow oscillations in anesthetized mice have been reported to range from 0.6 to 1.2 Hz (averaging 0.9 Hz possibly due to the different type of anesthesia)^8^.

Interestingly, we found that doubling the frequency of slow waves for a month elevated amyloidosis by increasing Aβ production. Acute pharmacological increases in synaptic activity have been shown to increase APP processing and Aβ production^27^. Furthermore, optogenetic activation that led to long-lasting neuronal hyperexcitability in the hippocampus also augmented Aβ pathology in AD mice^18^. However, epileptic seizures that were elicited in the animals might have accounted for the increased amyloidosis. In contrast, no overt seizures were observed when the frequency of slow waves was doubled in our study underlining the fact that a slight increase in frequency was within the physiological range of the circuit. Since the sleep-wake patterns were not monitored in these animals, the effect of stimulation on such is yet to be determined. Furthermore, the effect of light stimulation with duration of 400 ms on neuronal fatigue is not clear.

Another example of neuronal activity disruptions are gamma oscillations, which are important during sharp-wave ripples^14^. Restoration of gamma activity patterns by optogenetic targeting of a specific inhibitory interneuron class decreased amyloid beta production and amyloid deposition in AD mice^15^. Optogenetic stimulation using a random activity pattern failed to ameliorate amyloidosis despite the fact that the same amount of stimulation was applied^15^. Similar to the results in the present study, maintaining the activity pattern of the circuit was necessary to restore its proper function. Thus, regardless of targeting the excitatory or the inhibitory elements of a given circuit, as long as the normal activity pattern was recovered, so was the neuronal function. Restoration of normal circuit function slowed the disease progression.

Calcium plays a major role in maintenance of proper neuronal function^28^. Thus, calcium homeostasis is tightly regulated within neurons. Calcium homeostasis is disrupted in AD^20,29,30^. Restoration of slow wave power led to amelioration of calcium dyshomeostasis, evident by decreases in the number of neurons exhibiting calcium overload^2^. Interestingly, doubling the slow wave frequency exacerbated neuronal calcium dyshomeostasis in neuronal processes and somas, the two cellular compartments where calcium has differential functions. Thus, speeding up slow wave activity affected neurons directly by elevating their baseline calcium levels likely caused by elevations in soluble Aβ^25^. Additionally, APP mice exhibited decreased spine density on their cortical dendrites possibly due to overexcitation of the circuitry. Doubling slow wave frequency decreased the spine density further, possibly highlighting a compensatory mechanism to alleviate hyperactivation of the circuit.

AD is a progressive disorder. Disruptions of inhibitory elements of the circuit were evident in young mice. Specifically GABA, GABA_A_ and GABA_B_ expression was downregulated in cortices of young APP mice^2^. Here we report that levels of the excitatory neurotransmitter glutamate decreased after the start of amyloidosis. Doubling the slow wave frequency failed to further decrease levels of GABA, glutamate and GABA receptors possibly due to a floor effect.

A number of recent clinical trial failures underscores the need for development of AD therapeutics with targets additional to Aβ^31,32^. Neuronal circuitry disruptions provide an opportunity to consider targeting excitatory and/or inhibitory elements of the circuits. Use of brain stimulation methodologies, such as transcranial magnetic stimulation (TMS) and transcranial direct current stimulation (tDCS) pose great promise. However, it is important to consider the stage of the disease progression at which to intervene. Also, stimulation parameters and the specific brain regions targeted will have to be carefully selected to maximize the benefit to risk ratio. This and earlier studies^2^ suggest that targeting circuitry early, prior to amyloidosis onset is the most efficient way to halt the disease progression. Furthermore, the power and the frequency of a neuronal activity pattern have to be mimicked with great care for a given circuit, since stimulations at other patterns might prove detrimental rather than beneficial. Thus, restoration of neuronal activity is a promising treatment option to pursue in AD patients, similar to those with other disorders. However, further studies are needed to test whether restoring circuit dynamics slows disease progression in AD patients.

## Methods

### Animals

The APPswe/PSdE9 transgenic mouse line was used in the present study. The mice overexpressed the amyloid precursor protein with the Swedish mutation as well as the deltaE9 mutation in presenilin 1 (APP mice)^33^ (Stock #034829, The Jackson Laboratory). Age matched non-transgenic littermates were used as controls (WT mice). Animals were 3–9 months of age unless specified otherwise. The studies followed Massachusetts General Hospital (MGH) Animal Care and Use Committee and NIH guidelines for the use of experimental animals. The MGH Institutional Animal Care and Use Committee (IACUC) approved this study. Males and females were used in this study. Animals of the same sex were housed up to 4 animals/cage. Individual housing was maintained subsequent to surgical procedures. Animals were housed in microdialysis bowls during chronic optogenetic treatments. All animals had free access to water and food ad libitum. Animals were maintained on a 12/12 hour day/night cycle in pathogen-free environment.

### Voltage-sensitive dye (VSD) imaging

VSD imaging was performed as described previously^2^. 1–2% isoflurane was used to anesthetize the animals. After being placed in a stereotaxic apparatus, heating pad was used to maintain animal’s body temperature throughout the length of anesthesia. Each animal’s scalp was disinfected with betadine followed by isopropyl alcohol. An incision was made. A round 5–8 mm craniotomy was performed over somatosensory cortex. After dura removal, 1 mg/ml Rh1691 in phosphate-buffered saline (PBS) (Optical Imaging) was applied directly to the brain for 1 hour. Then the cortex was washed with PBS to remove unbound dye. A 5–8 mm glass coverslip was attached to the surrounding skull using dental cement and Krazy glue.

Wide field VSD imaging was carried out using a 2X long working distance objective (NA = 0.14) on an upright microscope (BX51, Olympus). 120 W mercury arc lamp was used to excite the VSD with 585/20 nm light. Hamamatsu ORCA-ER digital CCD camera was used to collect images with 5 ms exposure every 50 ms. Image sequences were acquired in 50 s epochs, up to 10 epochs/condition.

### Electrophysiological recording

Three weeks after ChR2 virus injection in the left anterior cortex, an optical fiber was implanted at the virus injection site (DV 1.0 mm), and a probe consisting of five tungsten stereotrodes was implanted in the contralateral anterior cortex (DV −0.3 mm to −0.8 mm, relative to dura, positioned to the center of the typical VSD imaging field of view). A skull screw was inserted over the cerebellum to serve as reference. After one week of recovery, electrophysiological recordings were acquired in the home cage. Local field potentials (32 kHz sampling, 0.1–300 Hz) were recorded from each contact site on the probe (Neuralynx^TM^). After a 10-min baseline recording, 10-min of laser illumination (473 nm, 1.2 Hz, 400 ms, 10–20 mW) was applied to the left cortex through the optic fiber linked to the implanted cannula via a sleeve (Doric Lenses). A TTL signal was used to identify laser ON times. Matlab (MathWorks, Natick, Massachusetts) was used for data analysis. Spectral analyses were performed using the Chronux toolbox http://chronux.org/. Power comparisons were made exactly at 1.2 Hz.

### Virus injections

Intracortical viral injections were made as described previously for optogenetics experiments^2^. Briefly, animals were anesthetized and their scalp disinfected. An incision was made in the skin down the center. Intracortical virus injections were made. Left anterior cortex was injected with a virus containing 1.5 μl of AAV5-CamKIIα-hChR2(H134R)-mCherry (3 × 10^12^ virus molecules/ml) or a control vector AAV5-CamKIIα-mCherry (University of North Carolina) with the following coordinates: AP +1, ML +0.5, DV −1 at 0.13 µl/min. Yellow Cameleon 3.6 (YC3.6) is a ratiometric probe that allows visualization of subcellular calcium dynamics, which are disrupted in AD^20,34^. Right posterior cortex was injected with a viral vector containing 1.5 μl of AAV2-CBA-YC3.6 (University of Pennsylvania) with the following coordinates: AP −3, ML −1, DV −0.7 at 0.13 µl/min (2 × 10^12^ molecules/ml). The skin was then sutured and virus was allowed to express. 3 weeks later, craniotomies were performed over the right hemisphere. Expression of YC3.6 was verified and imaged in anesthetized mice.

For microdialysis experiments, the right anterior cortex was injected with 1.5 μl of AAV5-CamKIIα-hChR2(H134R)-mCherry (3 × 10^12^ virus molecules/ml) virus with the following coordinates: AP +1, ML −0.5, DV −1 at 0.13 µl/min. Microdialysis probe was installed in the left hemisphere (see below). mCherry expression was verified in all post-mortem brains included in this study.

### Light activation of ChR2

The frequency of slow oscillations is ~0.6 Hz in APP mice and nontransgenic controls^2^. To determine whether light activation of ChR2-expressing pyramidal neurons is able to elicit slow oscillations at twice the normal frequency, the animals were subjected to an acute optogenetic treatment at 1.2 Hz (twice the endogenous frequency of slow oscillations) under isoflurane anesthesia. To that end, a light-guide cannula (Doric Lenses) was situated over the ChR2 injected site. The cannula tip was positioned above the cortex to prevent disturbance of the cortical neuronal networks. A fiberoptic was attached to the cannula via a sleeve (Doric Lenses). 400 ms pulses of 473 nm light (10–20 mW) from a diode laser (Optoengine) were applied to the cortex at 1.2 Hz, twice the endogenous frequency of slow oscillations. Similarly, light activation of ChR2-expressing cortical neurons at 1.2 Hz was used during microdialysis while animals were awake and freely moving in microdialysis bowls (Harvard Apparatus) with free access to food and water.

Optogenetic treatment was used to drive slow oscillations in mice continuously from 4 to 5 months of age by synchronously stimulating excitatory cortical neurons with light activation of ChR2. 400 ms pulses of 473 nm light (10–20 mW) from a diode laser (Optoengine) were applied to the cortex via a fiber optic at 1.2 Hz, 24 hr/day for 30 days (1 month). Freely moving animals were maintained in microdialysis bowls (Harvard Apparatus) with free access to food and water for the duration of chronic treatment. Subsequently amyloid and calcium levels were determined using multiphoton microscopy.

Nontransgenic controls were used to determine whether driving bursting activity at twice the frequency would elevate intracellular calcium independent of APP and PS1 overexpression. Month-long light activation of ChR2 that elicited slow oscillations at twice the frequency was performed on wildtype animals from 4 to 5 months of age. Intracellular calcium levels were determined after imaging YC3.6. To control for light toxicity, month-long light treatment was performed in APPs and WT animals expressing the control construct (mCherry only, lacking ChR2).

### Multiphoton imaging and data acquisition

Amyloid plaque and YC3.6 imaging was performed as described previously^2,35–37^. One day before multiphoton imaging, animals were intraperitoneally injected with methoxy-XO4 (10 mg/kg) to allow visualization of amyloid plaques. 1–2% isoflurane was used to anesthetize the mice on the imaging day. Imaging of amyloid plaques and YC3.6-expressing neurons was carried out with a commercial multiphoton system (Olympus Fluoview 1000MPE) mounted on an Olympus BX61WI upright microscope. Image acquisition was performed using a 25X water immersion objective (NA = 1.05, Olympus). A mode-locked titanium/sapphire laser (MaiTai; Spectra-Physics, Fremont, CA) was used to generate two-photon fluorescence with 800 nm or 860 nm excitation. Detectors consisting of three photomultiplier tubes (Hamamatsu, Ichinocho, Japan) collected light in the following ranges: 380–480, 500–540, and 560–650 nm. Amyloid plaques were imaged using 800 nm excitation at 1X optical zoom^38^. YC3.6-expressing neuronal processes (neurites) and cell bodies were imaged with 860 nm excitation at 5X and 2X optical zoom respectively^20^. Dendritic spines were imaged at 5X zoom. Laser power was maintained below 50 mW at the objective to avoid phototoxicity. 1–5 μm steps were used to acquire Z-stacks of amyloid bearing plaques and calcium-expressing neurons. At the end of imaging session, CO_2_ was used to euthanize the mice following by perfusion with phosphate buffered saline (PBS). Once brains were isolated, one hemisphere was fixed in 4% paraformaldehyde with 15% glycerol cryoprotectant for 24 hours, frozen in mounting media on ice cold isopropanol, sectioned into 20 μm-thick slices on a cryostat (Leica) and mounted onto glass slides. The second hemisphere was flash-frozen in liquid nitrogen and saved for HPLC.

### Image processing, data analysis

#### VSD image analysis

ImageJ (http://rsbweb.nih.gov/ij/) was used to analyze VSD image sequences of cortical activity. Each raw image sequence was processed in the following way. A dF/F0 image sequence was calculated as pixel intensity (dF) divided by the lowest image fluorescence intensity in the sequence (F0). Displayed VSD traces represent mean dF/F0 values within a somatosensory cortical region of interest (ROI) <5–8 mm. Fourier transform analysis using Matlab was used to determine oscillation powers and frequencies. Matlab was also used to generate the power spectral density plots.

#### Amyloid plaque and YC3.6 analysis

Images acquired with multiphoton microscopy were analyzed in ImageJ. Amyloid plaque analysis was performed in the following way. Maximum intensity projections of each z-stack containing amyloid plaques were generated. Then amyloid plaques were manually counted and measured. Data were represented as amyloid plaque number per respective cortical volume. To determine amyloid plaque burden, each projected image was thresholded and segmented. Calculation of the percentage area occupied by amyloid was made. The signal from cerebral amyloid angiopathy, or CAA (amyloid lining the vessels), was excluded. Data were represented as amyloid plaque burden per respective cortical volume.

Images of YC3.6-expressing neurons were also analyzed with ImageJ. Since YC3.6 is a ratiometric probe, ratio images of YFP/CFP were created as follows. The background, calculated as the mode at the last image of each volume, was subtracted from each image in the stack and a median filter with radius 2 was applied to YFP and CFP images. Then the emitted fluorescence intensity of YFP was divided by CFP. ROIs of neuronal processes (neurites) and cell bodies were selected using the YFP images manually in ImageJ. YFP/CFP Ratios were determined for each ROI. YFP/CFP ratios were converted to intracellular calcium concentrations, [Ca^2+^]I, with standard ratiometric equations using the *in situ* Kd and Hill coefficient of YC3.6 as done previously^20^. Matlab was used to create pseudocolored images based on the YFP/CFP ratios. Absolute calcium concentration were determined by converting the YFP/CFP ratios using the empirical Rmin and Rmax and assigned to the jet colormap. The YFP/CFP ratio values were used to assign the Hue and Saturation (color) and the reference image was used to assign the Value (intensity). Data is represented as histograms and percentage of neuronal processes or cell bodies exhibiting calcium elevations or overload.

#### Spine density analysis

Image stacks acquired at 5X zoom were used for spine density analysis. Dendrites were identified and outlined. Spines present on dendrites were manually counted per each outlined dendrite using ImageJ. Spine density was calculated as number of spines divided by corresponding dendrite length. Dendrite segments ranging from 8–60 µm were included in the analysis.

### *In vivo* microdialysis and ELISA

*In vivo* microdialysis sampling of brain interstitial spinal fluid (ISF) Aβ was performed as described previously^39^. The microdialysis probe with a 4-mm shaft and a 3.0-mm, 1000-kDa-MWCO polyethylene membrane (PEP-4-03; Eicom, Kyoto, Japan) was used. Prior to use, the probe was conditioned in ethanol, and washed with filtered artificial cerebrospinal fluid (aCSF) perfusion buffer (in mM: 122 NaCl, 1.3 CaCl_2_, 1.2 MgCl_2_, 3.0 KH_2_PO_4_, and 25.0 NaHCO_3_). The probe’s outlet and inlet were connected to a peristaltic pump (ERP-10; Eicom) and a microsyringe pump (ESP-32; Eicom), respectively, with fluorinated ethylene propylene (FEP) tubing (φ 250 µm i.d.). Probe implantation was performed as described previously^39^, with minor modifications. Briefly, the animals were anesthetized with 1–2% isoflurane. A guide cannula (PEG-4; Eicom) was stereotactically implanted in the cortex (AP −2.0, ML 3.1, DV 0.7) and fixed with binary dental cement at 30° angle. Fiberoptic cannula was installed over the site expressing ChR2 (AP +1, ML −0.5, DV −1). 3 days later each mouse was placed in a separate standard microdialysis bowl. Microdialysis probe was inserted through the guide, fiberoptic was attached to the cannula. To obtain stable recordings, the probe and connecting tubes were perfused with aCSF for 240 min at a flow rate of 10 µl/min prior to sample collection. Samples were collected hourly at a flow rate of 0.1–1.0 µl/min before, during, and after light stimulation at 1.2 Hz as described above. Microdialysis samples were stored at 4 °C in polypropylene tubes. During microdialysis sample collection and optogenetic stimulation, mice were awake and freely moving in the microdialysis bowls, designed to allow unrestricted movement of the animals without applying pressure on the probe assembly (AtmosLM microdialysis system; Eicom). Total Aβ concentrations in microdialysis samples were assessed using BNT77-BA27 ELISA (Wako Pure Chemicals Industries).

### Immunohistochemistry

Standard immunohistochemical procedures were used. Transverse sections of mouse brains sliced at 20 µm thickness were incubated with the following antibodies: GABA (rabbit anti-GABA, 1:500, Sigma), GABA_A_ (rabbit anti-GABA_A_, 1:100, Millipore), GABA_B_, (guinea pig anti- GABA_B_R2, 1:500, Chemicon) and glutamate (rabbit anti-glutamate, 1:500; Abcam). Following 2 hour incubation with the primary antibodies at room temperature, the incubation with the appropriate secondary antibodies were performed for 1 hour. Then the sections were mounted with ProLong antifade reagent (Invitrogen). Each set of immunohistochemistry was performed on all tissue sections concurrently to control for variations in processing and allow quantitative analysis of staining intensity. ROIs were manually selected around individual cell bodies and/or processes. Optical density was calculated as a difference in ROI intensity and average intensity of the entire image divided by average intensity of the image, and represented as a percentage.

### High-performance liquid chromatography (HPLC)

A reverse-phase isocratic system containing an ESA model 584 pump, two 5011A ESA coulometric cells, a Dionex Ultimate 3000 autosampler and a model 5600A CoulArray detector (Thermo Electron North America LLC) was used for HPLC analysis. The following settings for the first and second electrodes in the series were used: +150 mV and +550 mV respectively. A batch-tested Waters Xterra^TM^ MS (3.0 × 50 mm) column at a flow rate of 0.3 mL/min was used for analyte separation. Fischer Scientific supplied all HPLC-grade chemicals. The mobile phase consisted of 100 mM sodium phosphate dibasic, 20% methanol, 3.5% acetonitrile. 85% phosphoric acid was used to bring the mobile phase to pH 6.7. Standard curves were made by serially diluting analyte stock solutions in 0.05 M perchloric acid. HPLC-grade water was used to prepare 100 µM Glutamate and GABA stock solutions. Standard curves were generated fresh for each run. Cortical samples were isolated from newly sacrificed animals and maintained at −80 °C until processing for HPLC analysis. The samples were homogenized in 400 µL of mobile phase and centrifuging at 13000 rpm for 15 minutes. Supernatants were isolated, filtered through a 0.2 µm Spin-X® filter tube (Corning® Costar®), and injected into the HPLC system.

Since Glutamate and GABA are not electrochemically active, a pre-column derivatization procedure using the classic, fast and stable amino acid derivatization protocol involving o-phthaldialdehyde (OPA) and beta-mercaptoethanol (ß-ME) was developed. 10 mL of derivatization stock was prepared by combining 27 mg OPA in 1 mL of methanol, 5 µL of ß-ME and 9 mL of 0.1 M sodium tetraborate, pH 9.3. Tetraborate was spun on a hot plate for 45 minutes at 100 °C and dissolved (pH 9.3). The derivatization stock solution was maintained at room temperature and shielded from light for up to 5 days. A working derivatization solution was prepared fresh from the stock for each run. To make the working derivatization solution, 2.5 mL of the derivatization stock was prepared in 7.5 mL of 0.1 M tetraborate at pH 9.3. The working derivatization solution was maintained at room temperature and shielded from light. The derivatization procedure was performed as follows. 15 µL of the working derivatization solution was added to 20 µL of sample, mixed, followed by a 1 minute incubation period prior to 27 µL of solution being sent over the column.

### Experimental design and statistical analysis

The present study aimed to investigate the importance of frequency when restoring slow oscillation activity in an APPswe/PS1dE9 mouse model of AD. Slow oscillations in the cortices of these mice and their wildtype littermate controls were detected with the voltage sensitive dye RH1691. Fourier transform analysis was executed to determine the nature of slow oscillation perturbations in transgenics and wildtype mice. The frequency of slow waves was doubled with light activation of ChR2. Animals were randomly assigned to groups for all experiments. Acute microdialysis was performed before, during and after optogenetic stimulation of wildtype and APP mice. For the chronic experiment, mice were assigned to a treatment (2X Rx) or a control group at 4 months of age and underwent a light treatment from 5 and 6 months. Amyloid plaque load and intraneuronal calcium levels were assessed *in vivo* using multiphoton imaging. Analyses included only the animals that recovered from surgical procedures and survived until the end of the experiment. Experimenters were kept blind to the nature of experimental conditions until statistical analyses. Immunohistochemistry was performed to determine protein levels of inhibitory and excitatory neurotransmitters GABA and glutamate as well as GABA_A_ and GABA_B_ receptors in cortical tissue. HPLC analyses were performed to verify cortical GABA and glutamate levels.

GraphPad 5.0 was used to perform statistical analyses. Data was represented as mean ± SEM. First, data were tested for normality (using Shapiro-Wilk normality test, D’Agostino & Pearson omnibus normality test or Kolmogorov-Smirnov test). Then appropriate statistical tests were implemented (student t test or one-way ANOVA for normally distributed data, Kruskal-Wallis test for nonparametric data). p < 0.05 was considered significant for datasets comparing 2 conditions. For datasets comparing 3 conditions or 4 conditions, p < 0.025 or p < 0.0125 respectively was considered significant.

## Supplementary information

Supplementary Information in 1 file
