# Supplementary material for: Frequency-dependent exacerbation of Alzheimer’s disease neuropathophysiology
Source: Sci Rep. 2019 Jun 20;9:8964. doi: 10.1038/s41598-019-44964-z (PMC6586873; doi:10.1038/s41598-019-44964-z)
Supplement: Supplementary file 1 — Supplementary Information in 1 file [file 41598_2019_44964_MOESM1_ESM.pdf]

Title:

Frequency-dependent exacerbation of Alzheimer's disease neuropathophysiology

Authors:

Ksenia V. Kastanenka*, Maria Calvo-Rodriguez, Steven S. Hou, Heng Zhou, Shuko Takeda, Michal Arbel-Ornath, Amanda Lariviere, Yee Fun Lee, Alex Kim, Jonathan M. Hawkes, Robert Logan, Danielle Feng, Xiqun Chen, Stephen N. Gomperts, Brian J. Bacskai*

Supplementary Figure 1. Effect of 2X Rx optogenetic stimulation on cortical LFP power in APP mice. ***A***, Top: LFP traces from one mouse. Blue, 473 nm laser pulses (1.2 Hz, 400 ms duration). Bottom: LFP power spectra from this mouse. The vertical arrow identifies the stimulation frequency. Blue: spontaneous LFP; Red: laser stimulation (2X Rx). Data are smoothed with a 25 ms gaussian for display. ***B***, LFP power spectra across the 3 APP mice (no smoothing). Note the impact on power at 1.2 Hz. ***C***, Group level effect of optogenetic stimulation on LFP power at 1.2 Hz. Data are shown as mean ± SEM (n = 3 mice, p = 0.017, paired t-test).

Supplementary Figure 2. Increasing slow wave frequency elevates amyloid plaque load in APP mice. ***A-C***, Multiphoton microscopy images of methoxy-XO4 positive plaques at 9 months of age in a control APP mouse (***A***, control), in an APP mouse undergoing light activation of mCherry lacking ChR2 (***B***, mCherry 2X Rx), and an APP mouse whose frequency of slow waves was increased with light activation of ChR2 (***C***, ChR2 2X Rx). Scale bar, 30 µm. ***D-E***, Bar graphs representing amyloid plaque number (***D***) or amyloid plaque burden (***E***) per cubic millimeter of mouse cortex (n=4-5 mice/group). Data are shown as mean ± SEM. N.s. not significant. *** p<0.001.

Supplementary Figure 3. Increasing the frequency of slow waves elevates ISF Aβ in APP mice. ***A***, Schematic of mouse brain undergoing light stimulation of ChR2-mCherry expressed in right cortex and microdialysis through the microdialysis probe installed in the left cortex. ***B,*** Experimental protocol. Arrows indicate stimulation of slow oscillation circuit by light activation of ChR2 to increase the frequency of slow waves by a factor of 2. ***C-D,*** Levels of ISF Aβ before, during and after stimulation in the wildtype (***C***) and APP (***D***) mice (n=3-6 mice/group). Data are shown as mean ± SEM. *p<0.05. ** p<0.01.

Supplementary Figure 4. Inhibitory and excitatory neurotransmitter deficits in 9 month old APP mice. ***A-C***, GABA immunoreactivity in the cortices of 9 month old control (***A***) and treated (***B, C***)

wildtype mice. ***D-F***, GABA immunoreactivity in the cortices of 9 month old control (***D***) and treated (***E, F***) APP mice. Scale bar, 20 µm. ***G***, Bar graph depicting normalized fluorescence intensities of GABA immunoreactivity across conditions (n=4-9 mice/group). ***H***, Bar graph comparing cortical GABA levels measured with HPLC across conditions (n=4-11 mice/group). ***I-K***, Glutamate immunoreactivity in the cortices of 9 month old control (***I***) and treated (***J, K***) wildtype mice. ***L-N***, Glutamate immunoreactivity in the cortices of 9 month old control (***L***) and treated (***M, N***) APP mice. ***O***, Bar graph depicting normalized fluorescence intensities of glutamate immunoreactivity across conditions (n=4-9 mice/group). ***P***, Bar graph comparing cortical glutamate levels measured with HPLC across conditions (n=4-15 mice/group). N.s., not significant. * $p<0.05$, *** $p<0.001$.

Supplementary Figure 5. Inhibitory neurotransmitter receptor deficits in 9 month old APP mice. ***A-C***, $GABA_A$ immunoreactivity in the cortices of 9 month old control (***A***) and treated (***B, C***) wildtype mice. ***D-F***, $GABA_A$ immunoreactivity in the cortices of 9 month old control (***D***) and treated (***E, F***) APP mice. Scale bar, 20 µm. ***G-I***, $GABA_B$ immunoreactivity in the cortices of 9 month old control (***G***) and treated (***H, I***) wildtype mice. ***J-L***, $GABA_B$ immunoreactivity in the cortices of 9 month old control (***J***) and treated (***K, L***) APP mice. ***M***, Bar graph depicting normalized fluorescence intensities of $GABA_A$ immunoreactivity across conditions (n=4-9 mice/group). ***N***, Bar graph depicting normalized fluorescence intensities of $GABA_B$ immunoreactivity across conditions (n=4-9 mice/group). * $p<0.05$, *** $p<0.001$.

Supplementary Figure 6. Slow oscillation power before (Spontaneous) and during (2X Rx) optogenetic manipulation in wildtype and APP mice (n=3-4 mice/group). Data are shown as mean ± SEM. *$p<0.05$, ***$p<0.001$.

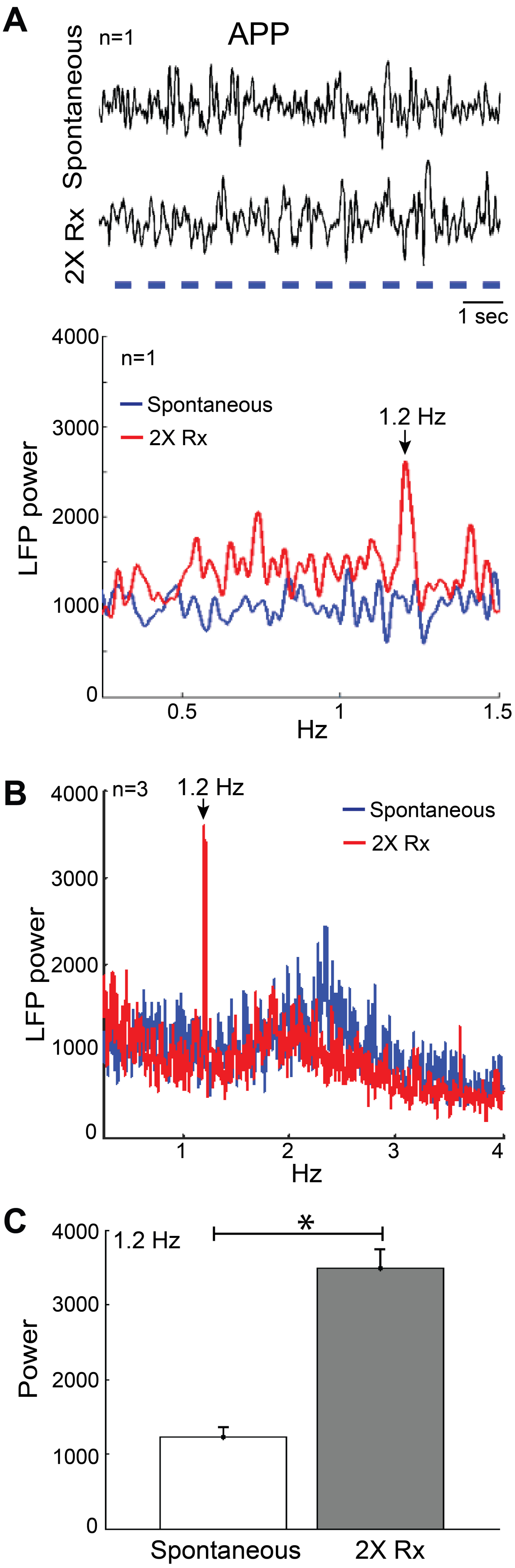

Supplementary Fig. 1

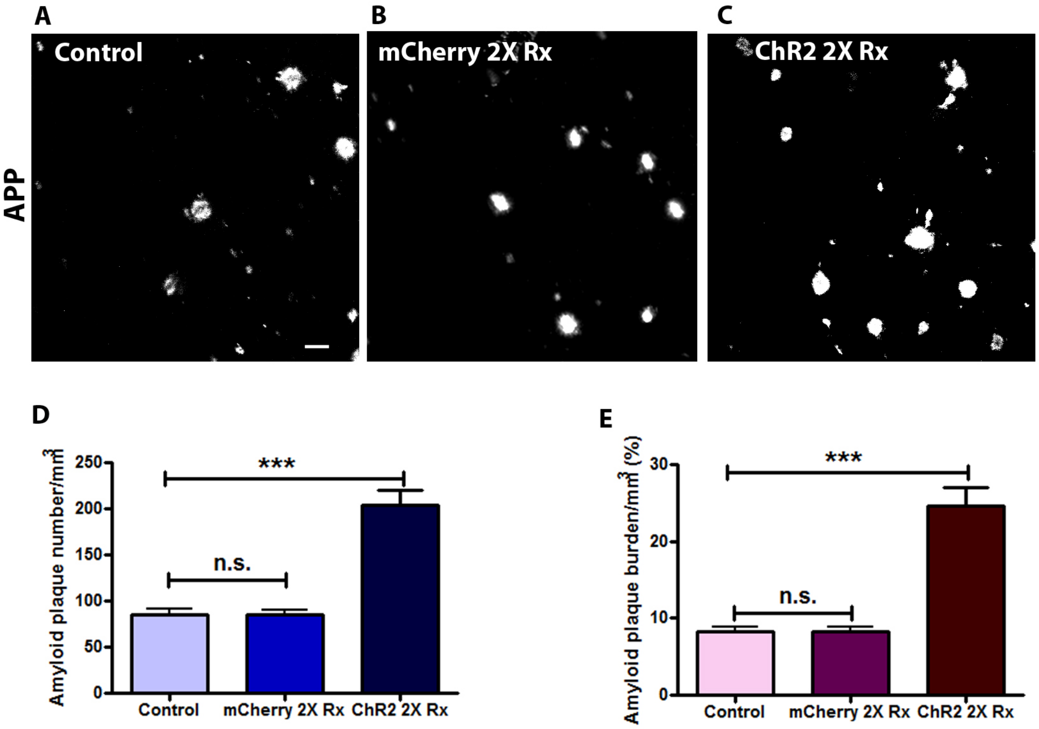

Supplementary Fig. 2

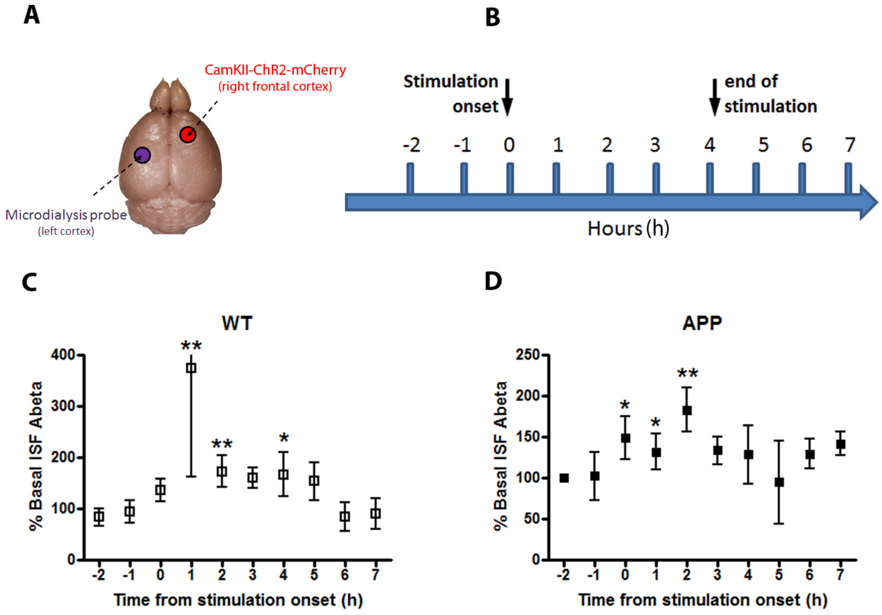

Supplementary Fig. 3

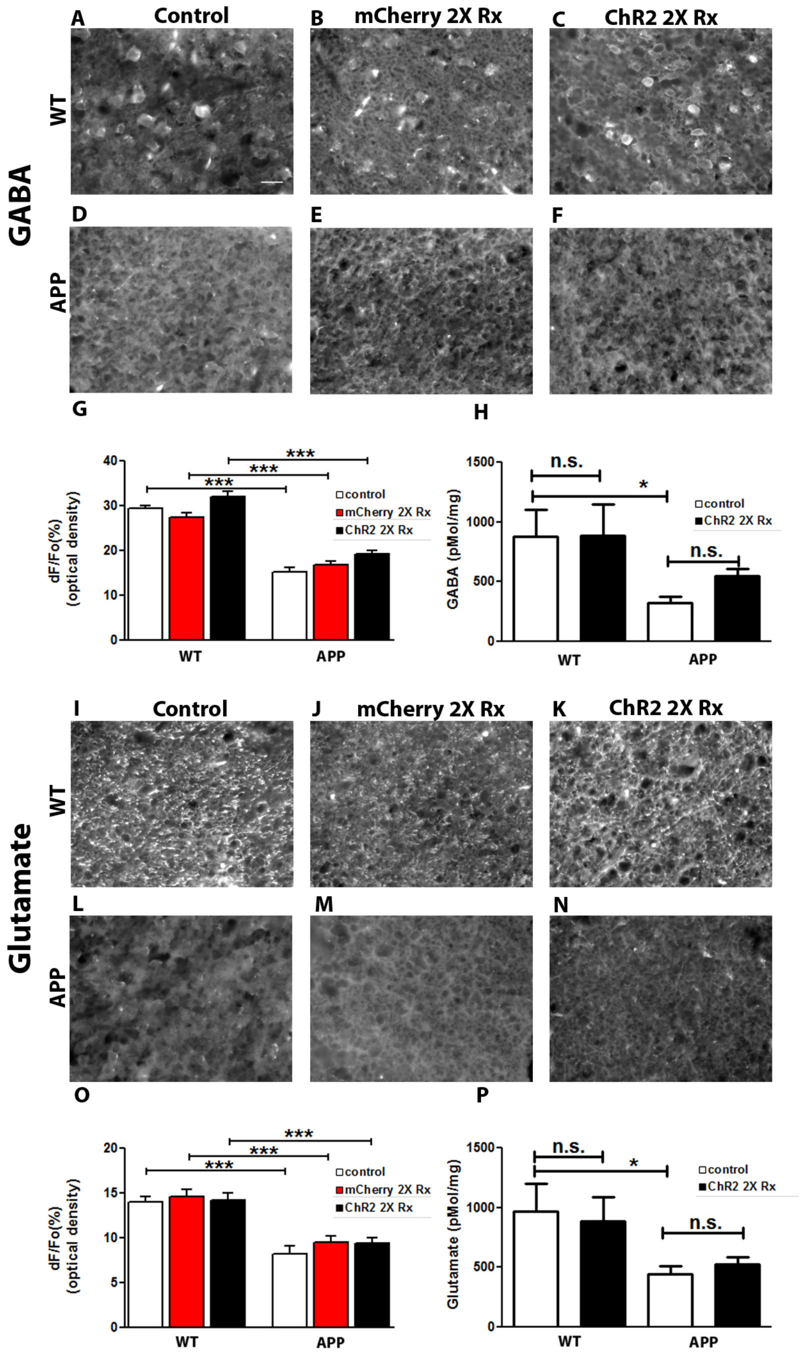

Supplemetary Fig. 4

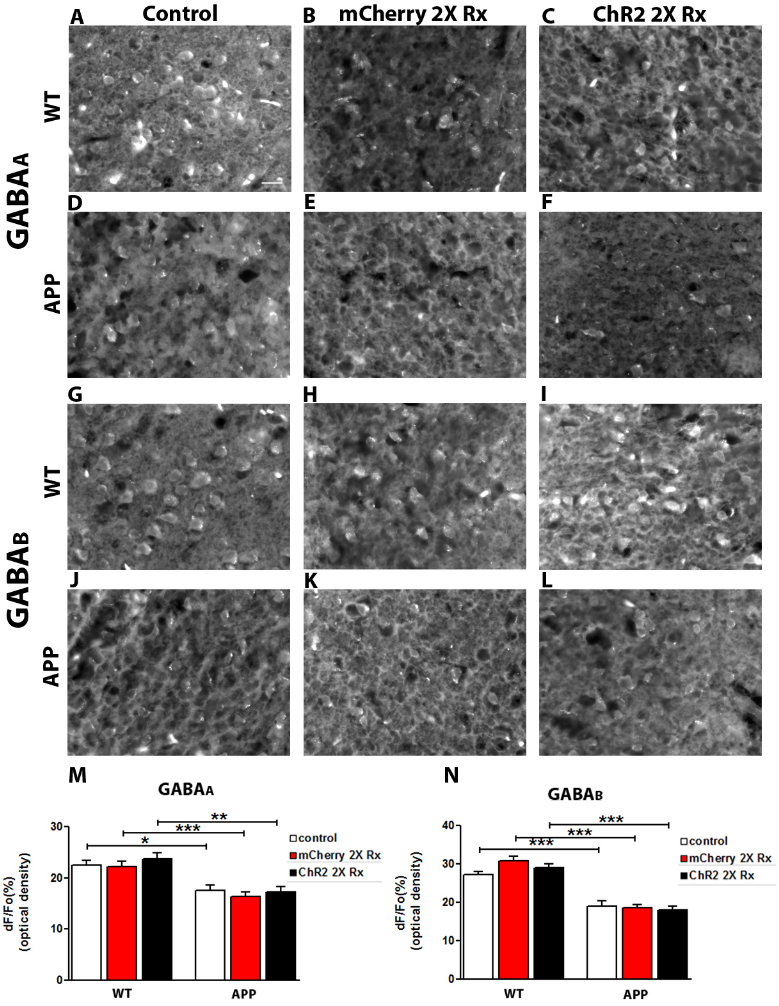

Supplementary Fig 5

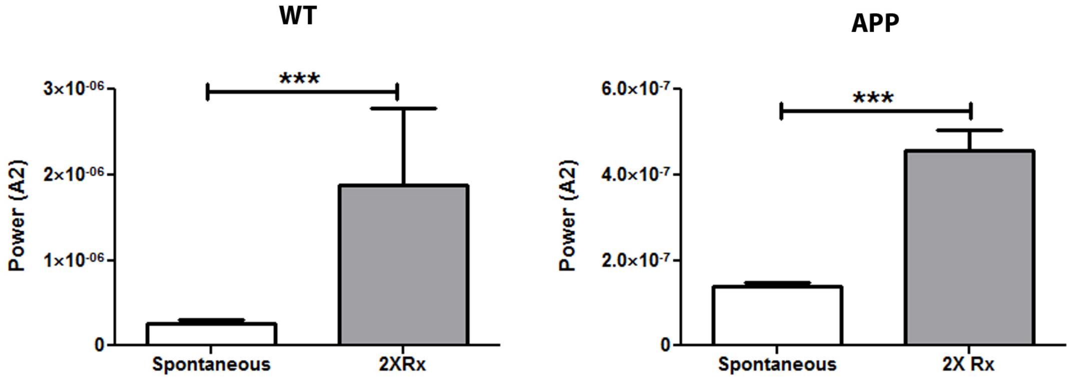

Supplementary Fig. 6
